# Objective Outcome Measures Continue to Improve from 6 to 12 Months after Conservatively Treated Distal Radius Fractures in the Elderly—A Prospective Evaluation of 50 Patients

**DOI:** 10.3390/jcm10091831

**Published:** 2021-04-22

**Authors:** Rikke Thorninger, Daniel Wæver, Jonas Pedersen, Jens Tvedegaard-Christensen, Michael Tjørnild, Martin Lind, Jan Duedal Rölfing

**Affiliations:** 1 Department of Orthopaedics, Randers Regional Hospital, 8930 Randers, Denmark; daniel_waever@yahoo.dk (D.W.); jonas2889@outlook.com (J.P.); jensch@rm.dk (J.T.-C.); Michael.tjornild@rm.dk (M.T.); 2 Department of Clinical Medicine, HEALTH, Aarhus University, 8000 Aarhus, Denmark; martinlind@dadlnet.dk (M.L.); jan.roelfing@clin.au.dk (J.D.R.); 3 Department of Orthopaedics, Aarhus University Hospital, 8200 Aarhus, Denmark

**Keywords:** distal radius fracture, fracture, non-operative treatment, conservative treatment, complications, patient-reported outcome measures, Quick-DASH, PRWHE, NRS, osteoporosis, aging

## Abstract

Distal radius fractures (DRF) in the elderly population above 65 years represent 18% of all fractures and are thereby the second most frequent fracture in the elderly. Fracture dislocation and comminution are often used to determine whether non-operative or operative treatment is indicated. The purpose of this prospective case series of minimally displaced DRF treated with a dorsal cast was to assess the complication rate and patient-reported outcome measures. This single-centre, single-blinded, prospective case series followed 50 conservatively treated DRF patients for one year. Primary outcomes were complications and Quick Disability of Arm Shoulder and Hand (qDASH) score. Secondary outcomes were range of motion (ROM), grip strength and pain, and Patient-Rated Wrist/Hand Evaluation (PRWHE). Results showed only minor complications with a return to prior ROM, qDASH, and pain after 12 months and improvement in outcomes after 6–12 months. In conclusion, the majority of DRF patients who were treated non-operatively with five-week dorsal casting recover fully after minimally displaced DRF. This standard approach is thus considered safe, and the present results provide a reference for other studies.

## 1. Introduction

Distal radius fractures (DRF) account for 18% of all fractures in the elderly ≥65 years of age and constitute the second most frequent fracture in the elderly next to hip fractures (37%) [1]. DRF are often low-energy fractures, predominantly occurring in females with an estimated life-time risk of DRF of 15% in contrast to a life-time risk of 2% in males [2]. The elderly population in Europe is estimated to increase by 56% in men and 41% in women within fifteen years, and therefore an increased incidence of DRF may be expected [3].

In the last two decades there has been a trend towards surgical intervention using volar locking plating (VLP). In Denmark, the treatment of a DRF is based on the National Clinical Guidelines (NCG) stipulated by The Danish Health Authority [4]. According to the NCG, a low-energy DRF, regardless of age, should be operatively treated when the fracture fulfills at least one of the following radiologic criteria after attempted closed reduction:

- >10° dorsal angulation of the radial joint surface;
- >2 mm articular step-off;
- >2 mm ulnar variance;
- Incongruence of the distal radioulnar joint;
- Substantial dorsal comminution indicating cross instability.

The reliability of these specific radiologic criteria has been questioned [5]. Internationally, different measurable radiological parameters have been reported to be of use in clinical decisions in predicting the functional outcome after a DRF, but the fundamental evidence verifying these parameters is varied and inconsistent [6,7].

Complications after DRF are not clearly defined, making it difficult to compare different complication rates in the literature. However, the literature points out that more complications may follow using VLP compared with conservative treatment [8].

This raises an interest in clarifying the complication rate and functional outcome of DRF—especially in the elderly who may not benefit from surgery in the long term [9].

The aim of this study was to investigate the complication rate, functional outcomes and patient-reported outcomes after conservatively treated, minimally displaced DRF that do not fulfil the criteria for operative treatment according to the NCG.

## 2. Materials and Methods

### 2.1. Study Design

A prospective case series of 50 DRF patients (≥65 years old) not fulfilling the NCG radiologic criteria for surgical treatment. The follow-up time was 12 months from the day of injury with out-patient visits after two weeks, five weeks, and six and 12 months. The study was conducted at Regional Hospital Randers, Denmark, with an estimated coverage area of 270,000 inhabitants. Recruitment took place from 1 November 2018 until 31 July 2019.

Inclusion and exclusion criteria are stated in the published study protocol [9]. In brief, all patients were 65 years old or older and had sustained a minimally displaced DRF that does not fullfill the NCG criteria for operative treatment (please refer to the introduction).

### 2.2. Recruitment

All participants’ eligibility was approved by a consultant of the research group or the house physician on call. Primarily, contact with patients was established in the emergency department (ED). The patients were informed orally and in writing about the study and invited to enroll by giving written consent. Each patient treated in the ED was discussed the following day, and all radiographs were evaluated at an ED conference. This ensured that every potential participant was assessed for eligibility and offered enrollment either directly in the ED or the following day by telephone.

### 2.3. Interventions

When a displaced DRF was diagnosed using standardized wrist radiographs with an anterior-posterior and lateral/axial projection, a 20 mg/mL Lidocaine hematoma block was induced. The physician on call had two attempts to perform closed reduction in order to achieve an acceptable radiologic reduction according to the NCG. The closed reduction and plaster immobilization were guided by fluoroscopy. The assessment of the closed reduction was based on new standardized radiographs in two projections obtained at the Department of Radiology. If the inclusion criteria were met, the patient was offered enrollment.

Furthermore, undisplaced and minimally displaced DRF fulfilling the NCG criteria for non-operative treatment were enrolled. The cast was removed after five weeks.

If reduction was lost at the two-week follow-up in the outpatient clinic and thus no longer fulfilled the radiologic NCG criteria for non-operative treatment, the participant was offered surgery and excluded from the present study.

#### 2.3.1. Primary Outcomes

Complications were reported by the patient on a standardized form. The treating physician furthermore examined the patient and qualified the patients’ responses and added additional observations. Thus, both patient-reported and objective complications were reported and registered. Complications were defined as one of the following:

- Sensory disturbance including carpal tunnel syndrome and chronic regional pain syndrome (CRPS);
- Flexor tendon rupture and irritation;
- Extensor tendon rupture and irritation;
- Infection: superficial or deep;
- Hardware failure and hardware loosening;
- Reoperation including hardware removal or replacement.

Furthermore, vascular compromise with a capillary refill of more than two seconds and a free text field was available on the form for reporting other complications or qualifying the complications mentioned above.

#### 2.3.2. Secondary Outcomes

The validated Danish version of the Quick Disabilities of the Arm, Shoulder, and Hand (qDASH) was used to assess the patient-reported level of functionality [10,11,12]. The minimally clinical important difference (MCID) was a 16 to 20-point difference in qDASH [10,13,14].

Active range of motion (ROM) of the wrist, i.e., wrist flexion, extension, pronation, supination, radial deviation, and ulnar deviation was measured with a goniometer by an independent, blinded observer. Furthermore, patients wore stockings on their wrists in order to conceal minor deformities, etc. The ROM of the contralateral wrist served as a reference.

Grip strength was measured using an electronic hand dynamometer (EH101 CAMRY, by Camry scale). Grip strength is given as the mean of three measurements on each side [15,16]. The MCID of grip strength is 6.5 kg [17].

The EuroQol 5 dimensions—3 levels questionnaire (EQ5D-3L) was reported at six and 12 months. It contains five items (mobility, self-care, usual activities, pain/discomfort, and anxiety/depression) ranging from level 1–3 in each item [18].

Fracture-specific pain at rest was reported on a numeric rating scale (NRS) ranging from 0 to 10 [12].

The validated Danish version of the Patient-Rated Wrist/Hand Evaluation (PRWHE) was applied as a self-reported assessment of five items on pain, 10 items on function, and two optional items on appearance of the hand [19].

The following baseline demographics were recorded: gender, age, side of DRF, hand dominance (right-handed, left-handed, ambidextrous), working status, American Society of Anesthesiologists Classification (ASA class 1–6 ranging from 1 healthy, 2 mild systemic disease, 3 severe systemic disease, 4 severe systemic disease that is a constant threat to life, 5 moribund, 6 brain-dead), smoking (cigarettes/day), alcohol consumption (units/week), and diabetes (yes/no).

The preinjured state of qDASH, pain, and complications questionnaire were administered based upon recall of the patient at the time of injury.

### 2.4. Data Management and Statistical Analysis

Data were managed in accordance with Good Clinical Practice guidelines. Physical material with patient-identifiable data and informed consent were physically stored in a locked room according to national legislation. Data were collected physically on paper and subsequently registered in a database using REDCap (vers. 10.0.2, Vanderbilt University, Nashville, TN, USA, 2021) [20]. If a participant did not show up for follow-up in the out-patient clinic, the data steering committee established contact by telephone and/or mail in order to ensure participant retention in the study and to complete follow-up. The data were only accessible for the data steering committee.

The complication rate is presented in % (n/50). qDASH was presented in score points with a mean difference and 95% confidence intervals (CI). ROM of the wrist is presented as mean degree of motion for each movement with range and mean difference between injured and contralateral side with 95% CI. Grip strength is presented as difference in kilograms with 95% CI. Pain is reported as mean NRS with 95% confidence intervals.

Mixed effects analysis with correction for multiple comparisons was applied to analyse the longitudinal change of the different outcome measures, e.g., qDASH, VAS, angulation, and ROM. Spearman’s correlation was applied to qDASH vs. PRWHE. EQ5D-3L are given as raw data and indices.

Statistical significance was declared when *p* ≤ 0.05. All tests were performed using Prism 9 for macOS (vers. 9.1.0, GraphPad Software, San Diego, CA, USA, 2021).

## 3. Results

**Figure 1:**
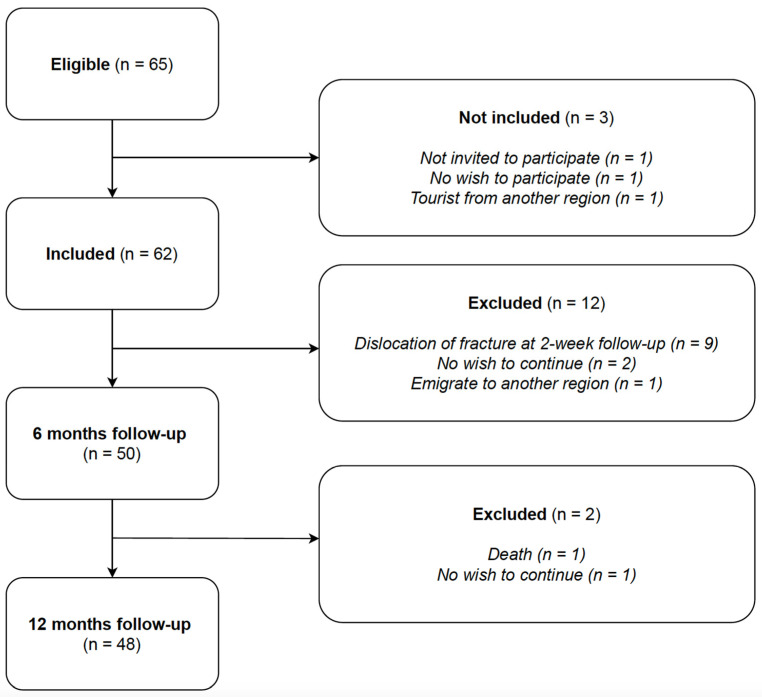
CONSORT Flowchart.

Figure 1 depicts the CONSORT flow diagram regarding eligibility, inclusion, and exclusion. In total, 50 patients were available for data analysis after six months follow-up and 48 patients after 12 months. Baseline demographics of the cohort are given in Table 1.

### 3.1. Primary Outcome Measure: Complications

8/50 (16%) reported complications after six months, while only 3/48 (6%) reported complications after 12 months. Here, two patients complained about sensory disturbances, and one patient complained about swelling during activity and lack of strength (Table 2). Table 2 also highlights the time dependency of sensory disturbances with six patients (12%) complaining about sensory disturbances after six months. However, none of these cases were motorically compromised, and no atrophy was observed. Thus, all sensory disturbances were classified as nerve irritation instead of, for instance, carpal tunnel syndrome. The complications registered as others were two cases of pain during activity.

### 3.2. Secondary Outcome Measures: Patient-Related Outcome Measures (qDASH and Their Correlation to PRWHE) and Pain Score (NRS)

**Figure 2:**
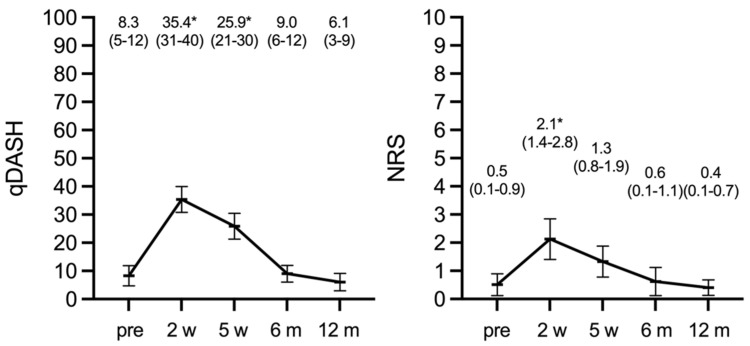
qDASH and NRS pain score, preinjury (pre), two weeks (w), five weeks (w), six and 12 months (m); * *p* < 0.05 compared with preoperative, i.e., recalled scores.

Both qDASH and pain score were statistically significantly worse at post-injury week two and five compared with the patient “recalled” scores before the injury (Figure 2). After six and 12 months, both outcome measures had returned to their preinjury level with no statistically significant difference between the three time points.

The change of mean PRWHE scores from 13.5 (95% CI 9.0–18.0, IQR 0–19) after six months to 8.7 (95% CI 3.6–13.7, IQR 0–10) after 12 months approached statistical significance (*p* = 0.05). To the PRWHE aesthetic item: “How important is the appearance of your hand to you?”, 41/50 patients responded not important, three patients somewhat important, and only one patient very important (five patients did not answer this question). Only the latter stated that the appearance of the wrist/hand bothered the patient significantly during the last week: 8 on a 0–10 Likert scale (not at all—worst possible).

Both patient-related outcome measure instruments had a strong correlation at any given time point: Spearmans r(PRWHE-qDASH) = 0.74 (*p* < 0.0001) after six months, and r(PRWHE-qDASH) = 0.66 (*p* < 0.0001) after 12 months. Furthermore, the correlation of the same instrument over time, e.g., from six and 12 months was also strong: r(PRWE(6 months–12 months)) = 0.50 (*p* < 0.0004), and r(qDASH(6 months–12 months)) = 0.56 (*p* < 0.0001).

**Figure 3:**
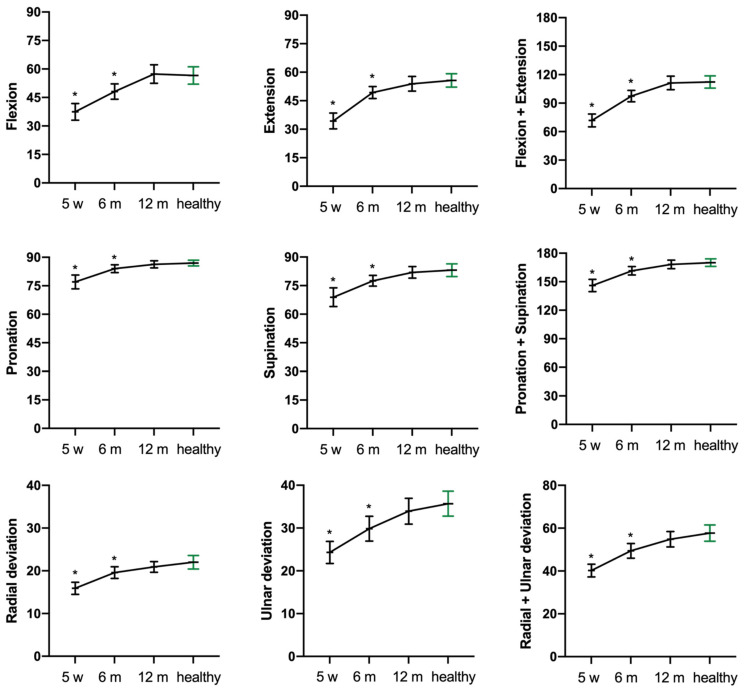
Temporal changes in active range of motion (degrees) after five weeks (w) and six and 12 months (m) compared with the healthy side at 12 months. * *p* < 0.05 compared with the healthy side (green).

Active ROM was still improving after six months and reached normal, i.e., contralateral ROM, after 12 months (Figure 3).

### 3.3. Grip Strength

The grip strength of the injured wrist increased statistically significantly from six to 12 months post injury (mean diff. 1.6 (95% CI 2.8–0.4, *p* < 0.01)). However, the grip strength of the injured side remained impaired compared with the uninjured side both at six months (mean diff. −6.0 (95% CI −7.9–−4.2), *p* < 0.0001) and 12 months (mean diff. −4.1 (95% CI −6.3–−1.9, *p* < 0.0001).

### 3.4. Quality of Life (EQ5D)

EQ5D-3L indices after six and 12 months were 0.87 (95% CI 0.84–0.90, range 0.68–1.00) and 0.93 (95% CI 0.90–0.96, range 0.71–1.00), respectively (*p* < 0.001). EQ-5D-3L frequency results reported by dimension (mobility, self-care, usual activities, pain/discomfort, anxiety/depression) are presented in Table 3.

### 3.5. Dorsal Angulation

The DRF of 27 patients was reduced using a hematoma block, correcting the mean angulation of 14.8° (95% CI 9.0–20.5) (*p* < 0.001). The mean dorsal angulation after reduction was 1.8° (95% CI −0.2–3.7). This correction was partially lost, i.e., 5.2° (95% CI 2.0–8.3; *p* = 0.001) during the five weeks of conservative treatment with a dorsal plaster cast.

**Figure 4:**
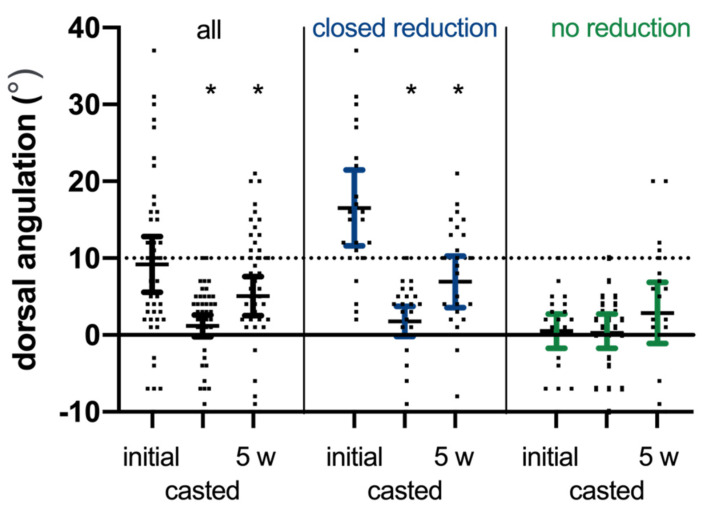
Dorsal angulation of the DRF at presentation, i.e., initial radiograph, the casted radiograph, and the final radiograph after five weeks (5 w) for all DRF and subdivided based on closed reduction (yes; no). * *p* < 0.05 compared with the initial dorsal angulation.

In the 23 patients without reposition, the mean dorsal angulation of 0.5° (−1.7–2.7) was maintained during treatment (mean difference: 2.4° (95% CI −0.2–4.9, *p* = 0.066), Figure 4). However, 9/27 reduced and 4/23 not-reduced fractures had a dorsal angulation of more than 10° on the latest radiographs after five weeks but had a dorsal angulation of less than 10° after the radiographical control two weeks post injury (Figure 4).

## 4. Discussion

The primary findings of the present study of 50 DRF patients with minimally displaced DRF treated conservatively with or without closed reduction and plaster immobilization was a low complication rate of 6% (3/48 patients) after 12 months. The complication types were sensory disturbances and activity-related wrist swelling. Interestingly, the reported complications were not consistent over time. After six months, 16% (8/50) of patients reported complications. However, the ulnar pain reported by two patients and the majority of sensory disturbances disappeared after 12 months. Please refer to Table 2 for details.

In agreement with these results, Saving et al. [15] investigated conservatively treated displaced DRFs after 12 months in elderly patients and found a complication rate of 11% consisting of five cases of nerve numbness and two cases of CRPS. Delayed extensor pollicis longus tendon rupture occurred in one of the cases within one year and up till 10 years after the fracture [21].

Subjective clinical outcomes based on the qDASH score improved statistical significantly from six to 12 months returning to preinjury levels. Contrary to our results, Aparicio et al. [22] found a significant increase in upper limb disability one year after the acquisition of conservatively treated DRF measured using the qDASH score. Dewan et al. [23] report that improvement in fracture-specific disability was completed after six months. This is in line with our results. However, we noticed a trend towards further improvement from six to 12 months (*p* > 0.05). qDASH as a tool is highly recommended for outcome measures in DRF [12]. In addition, the qDASH may even be more sensitive and responsive to functional impairments than the DASH (Disabilities of Shoulder and Hand) [24,25]. In our study, ROM also progressed from six until 12 months and normalized, which is corroborated by Hassellund et al. [26].

The findings of the present study confirms that closed reduction using a hematoma block is an acceptable and good treatment. Only 9/62 (15%) of the included patients did not maintain the reduction after two weeks and were thus excluded (Figure 1). However, mean change in dorsal angulation was 5.2 degrees (95% CI 2.0–8.3; *p* = 0.001) after the five weeks follow-up. Notably, 9/27 (33%) reduced and 4/23 (17%) non-reduced DRF had a dorsal angulation of more than 10° at the latest five-week radiographic follow-up. Nonetheless, the functional recovery and complication rate were not compromised in this group. Additionally, in this group there is a growing body of evidence in support of non-operative treatment in the long-term and a suggestion to reserve surgery for patients in need of fast recovery [15,26,27,28,29,30,31,32,33,34,35,36].

The included patients were relatively healthy (low ASA score) and had good preinjury function of the arm (low qDASH scores), thereby indicating a high demand for a good functional outcome. It is therefore encouraging that this was achieved despite 13/48 patients healed with a radiographic configuration normally mandating surgery according to the NCG [4].

### Strengths and Limitations

During the enrolment period only one potential study candidate missed inclusion; selection bias was therefore minimal. We only had few missing data: one patient died and one patient did not wish to participate; however, all patients had complete six-months data. The data collection of ROM was blinded, and the patients were instructed not to speak during the measurement. Limitations were the lack of a control group and the low ASA score in the study. Despite these strengths and limitations, we find that conservative treatment of DRF as described is to be considered a safe and reliable treatment for this group of patients.

## 5. Conclusions

In conclusion, in patients 65 years and older with conservatively treated non-displaced or minimally displaced DRFs, functional and patient-reported outcomes continue to improve from injury to six months and from six to 12 months. At the latest follow-up, the mean differences in qDASH, PRWHE, and ROM did not statistically significantly differ from the recalled preinjury or measured contralateral side.

## Figures and Tables

**Table 1:** Baseline Demographics.

		n (%)
Sex	Female	41 (82)
	Male	9 (18)
Age (years)	Median age	73.5
	Range	65–100
	IQR	70–78
Fractured side	Right	18 (36)
	Left	32 (64)
Hand dominance	Right	43 (86)
	Left	4 (8)
	Ambidextrous	3 (6)
	Dominant side fractured *	20 (40)
Working status	Full-time/part-time work	0 (0)
	Volunteer work	3 (6)
	Retired	47 (94)
Smoking status	Non-smoker	41 (82)
	Smoker	9 (18)
Alcohol consumption **	<7/14 units/week	44 (88)
	>7/14 units/week	6 (12)
ASA class	ASA class 1	16 (32)
	ASA class 2	25 (50)
	ASA class 3	9 (18)
	ASA class 4–5	0 (0)
Comorbidities	Osteoporosis	7 (14)
	Diabetes	3 (6)
	Hypertension	22 (44)
	Depression	9 (18)
Medications	No medications	5 (10)
	1–4 medications	38 (76)
	≥5 medications (polypharmacy)	7 (14)

* A fracture in an ambidextrous patient was not considered a fracture of the dominant side. ** Threshold defined as 7 units/week for females and 14 units/week for males.

**Table 2:** Complications.

Complications	Day 0 (n = 50)	2 Weeks (n = 50)	5 Weeks (n = 50)	6 Months (n = 50)	12 Months (n = 48)
Sensory disturbance	1	1	0	6 (12%) *	2
Flexor tendon rupture and irritation	0	0	0	0	0
Extensor tendon rupture and irritation	0	0	0	0	0
Vascular compromised (capillary refill ≥ 2 s)	0	0	0	0	0
Other	0	2 **	0	2 (4%) ***	1 ****
Total:	1	3	0	8 (16%)	3

* Five patients reported unspecific dysaesthesia of single digits, one hyperalgesia of the dorsal aspect of the wrist (4 × 10 cm); ** two patients had an exchange of dorsal plaster cast; *** two patients reported ulnar pain and pain during activity as complications; **** swelling during cycling and lack of strength.

**Table 3:** EQ-5D-3L frequencies reported by dimension and level after 6 month and 12 months, n (%).

Parameter	6 Months	12 Months
Mobility:		
Level 1	45 (90%)	39 (81%)
Level 2	5 (10%)	9 (19%)
Level 3	0 (0%)	0 (0%)
Total	50 (100%)	48 (100%)
Self-care:		
Level 1	45 (90%)	46 (96%)
Level 2	5 (10%)	2 (4%)
Level 3	0 (0%)	0 (0%)
Total	50 (100%)	48 (100%)
Usual activities:		
Level 1	43 (86%)	44 (92%)
Level 2	6 (12%)	4 (8%)
Level 3	1 (2%)	0 (0%)
Total	50 (100%)	48 (100%)
Pain/discomfort:		
Level 1	24 (48%)	39 (81%)
Level 2	26 (52%)	9 (19%)
Level 3	0 (0%)	0 (0%)
Total	50 (100%)	48 (100%)
Anxiety/depression:		
Level 1	46 (92%)	45 (94%)
Level 2	4 (8%)	3 (6%)
Level 3	0 (0%)	0 (0%)
Total	50 (100%)	48 (100%)

## Data Availability

The data presented in this study are available on request from the corresponding author. The data are not publicly available because the patients did not provide their written consent. If data are shared and used in other non-profit publications, this paper must be cited.
